# Pessimistic attributional style for positive life events as a predictor of low mental health in russian sample

**DOI:** 10.1192/j.eurpsy.2021.900

**Published:** 2021-08-13

**Authors:** T. Gordeeva, O. Sychev

**Affiliations:** 1 Psychology, Lomonosov Moscow State University, Moscow, Russian Federation; 2 Psychology, National Research University Higher School of Economics and Department of Psychology Moscow State University, Moscow, Russian Federation

**Keywords:** self-esteem, pessimistic attributional style, mental health

## Abstract

**Introduction:**

Attributional style (AS) indicates cognitive dispositions for explaining positive and negative events. People with pessimistic АS explain failure with stable and global causes. Previous studies and meta-analyses (Hu et al., 2015; Peterson et al., 1985; Zhang et al., 2014) showed that pessimistic AS for failures is a reliable predictor of depression and ill-being, but the possible mediators of such relations are understudied.

**Objectives:**

Our main objective was to analyse relations of pessimistic AS for success and failure with mental health. We hypothesized that pessimistic AS would be a predictor of low mental health mediated by self-esteem, dispositional optimism, and gratitude.

**Methods:**

A cross-sectional study was conducted on a sample of 261 adults (MA=32.09, SD=12.53, 13% male) using a 24-item attributional style questionnaire (SFASQ, Gordeeva et al., 2009), mental well-being scale (Tennant et al., 2007), self-esteem scale (Rosenberg, 1965), gratitude questionnaire (McCullough et al., 2002), and LOT (Scheier, Carver, 1985).

**Results:**

A path model of effects of pessimistic AS in positive and negative situations on mental ill-being was developed. The model with three mediators fits the data very well: CFI=0.990; RMSEA=0.048. The pessimistic attributional style for positive events was a significant predictor of mental ill-being mediated by self-esteem, dispositional optimism, and gratitude while the indirect effect of pessimistic AS for failures on mental ill-being (controlling for age) was not significant.

**Conclusions:**

Only the pessimistic AS for successes but not for failures was a significant predictor of mental ill-being which underline the importance of stable and global attributions of positive life events for mental health.

